# Characterization of natural co-cultures of *Piromyces* with *Methanobrevibacter ruminantium* from yaks grazing on the Qinghai-Tibetan Plateau: a microbial consortium with high potential in plant biomass degradation

**DOI:** 10.1186/s13568-017-0459-1

**Published:** 2017-08-07

**Authors:** Ya-Qin Wei, Hong-Jian Yang, Rui-Jun Long, Zhi-Ye Wang, Bin-Bin Cao, Qin-Chang Ren, Tian-Tian Wu

**Affiliations:** 1 Key Laboratory of Microbial Resources Exploitation and Application, Gansu Province, Institute of Biological Research, Gansu Academy of Sciences, Lanzhou, 730000 People’s Republic of China; 2 State Key Laboratory of Animal Nutrition, College of Animal Science and Technology, China Agricultural University, Yuanmingyuan West Road No. 1, Haidian District, Beijing, 100193 People’s Republic of China; 3 School of Life Sciences, Lanzhou University, Tianshui Southwest Road No. 222, Lanzhou, 730000 People’s Republic of China

**Keywords:** Yak, Anaerobic fungus, Methanogen, Lignocellulose, Bioconversion

## Abstract

Anaerobic fungi reside in the gut of herbivore and synergize with associated methanogenic archaea to decompose ingested plant biomass. Despite their potential for use in bioconversion industry, only a few natural fungus–methanogen co-cultures have been isolated and characterized. In this study we identified three co-cultures of *Piromyces* with *Methanobrevibacter ruminantium* from the rumen of yaks grazing on the Qinghai Tibetan Plateau. The representative co-culture, namely (*Piromyces* + *M. ruminantium*) Yak-G18, showed remarkable polysaccharide hydrolase production, especially xylanase. Consequently, it was able to degrade various lignocellulose substrates with a biodegrading capability superior to most previously identified fungus or fungus–methanogen co-culture isolates. End-product profiling analysis validated the beneficial metabolic impact of associated methanogen on fungus as revealed by high-yield production of methane and acetate and sustained growth on lignocellulose. Together, our data demonstrated a great potential of (*Piromyces* + *M. ruminantium*) Yak-G18 co-culture for use in industrial bioconversion of lignocellulosic biomass.

## Introduction

With energy consumption continuing to rise and fossil fuels inevitably trending toward limitation, humanity is urged to find alternative energy resources. One most recent emerging focus of energy generation has been the use of biofuels which can be generated from sustainable biomass feedstocks (Tilman et al. 2009). On the top of the list of renewable biomass resources that are suitable for biofuel production is crop straw, which is ranked as the fourth largest energy resources after coal, oil and natural gas. The major structural component of crop straw is lignocellulose, a heterogeneous complex mainly consisting of two carbohydrate polymers (cellulose, hemicellulose), and an aromatic polymer (lignin) (Bayer et al. 2004). Currently, the bioconversion strategy is regarded as the most common approach for industrial utilization of lignocellulosic biomass, which explores natural microbial colonizers of lignocellulose or their lignocellulose-degrading enzymes to decompose the recalcitrant structural polymers to easily metabolizable monosaccharides which are subsequently converted to products (Balan 2014). Improving the bioconversion efficiency of lignocellulosic biomass has received increased attention from researchers in recent years.

Anaerobic fungi isolated from rumen of ruminants are well known for their plant biomass-degrading capabilities (Theodorou et al. 1996). Despite only accounting for a very small percentage (<8%) of the rumen microbial community, anaerobic fungi are largely responsible for degrading lignin tissue (Gruninger et al. 2014). Their strong lignocellulose degradation capacity stems from both physical disruption by penetrating their rhizoids into the plant cell wall and enzymatic digestion by producing a wide range of fibrolytic enzymes to effectively break down lignocelluloses to monosaccharides (Boxma et al. 2004). Subsequently, the resulting monosaccharides are taken up by the fungi and utilized via two alternative metabolic pathways, the cytoplasmic pathway to produce formate, ethanol, lactate and succinate as end products, or the higher energy-yielding pathway in hydrogenosome to produce formate, acetate, CO_2_, H_2_ and ATP as end products (Muller 1993). The biodegradation capability of anaerobic fungi might be regulated by other members of rumen microbial community, as exampled by methanogens. Syntrophic relationship could form between anaerobic fungi and methanogens: methanogens can feed on some of the fungal metabolites including H_2_, CO_2_, formate to produce methane (CH_4_) (Bauchop and Mountfort 1981). Consequently, anaerobic fungi are often found to be physically associated with methanogens when isolated from herbivore rumen (Bauchop and Mountfort 1981; Jin et al. 2011; Leis et al. 2014). It was also demonstrated that artificial consortiums of rumen fungi and methanogens generally degrade fiber more effectively than the fungus mono-cultures (Bauchop and Mountfort 1981). Such enhanced biodegradation activity could be attributed to the change of fungal metabolism in the presence of methanogen: hydrogenosome metabolism is favored over cytoplasmic metabolism, and consequently more energy is produced for fungi to grow, resulting in accelerated lignocellulose fermentation kinetics (Marvin-Sikkema et al. 1993; Williams et al. 1994) Thus, the rumen-derived natural anaerobic fungus–methanogen consortiums hold substantial promise for application in industrial bioconversion of lignocellulosic biomass.

The domestic yak (*Bos grunniens*) is a large ruminant of the bovine family grazed mainly on the Qinghai Tibetan Plateau at an elevation higher than 3000 m. Given the facts that the yaks feed on wild grass instead of grain in order to thrive in the harsh environment and ruminal microbial community is critical for plant biomass digestion, we conceived the hypothesis that the natural fungus–methanogen co-cultures derived from the rumen of grazing yaks should evolve to acquire superior lignocellulose degradation ability as the result of long-term natural selection during the adaptation of yaks to the Qinghai-Tibetan Plateau. Our most recent study provided the first line of evidence supporting this hypothesis by isolating a total of 20 fungus–methanogen co-cultures from the rumen of the grazing yaks (Wei et al. 2016b). Among the three fungal genera (*Orpinomyces*, *Neocallimastix* and *Piromyces*) identified in these natural co-cultures, *Neocallimastix* and *Piromyces* genera are of particular interest as their monoculture isolates were previously demonstrated to produce high levels of fibrolytic enzymes (Paul et al. 2010). In the present study, we further investigated three *Piromyces* with *M. ruminantium* co-cultures isolated from grazing yaks. Moreover, we focused on studying the representative (*Piromyces* + *M. ruminantium*) Yak-G18 co-culture and provided a comprehensive, quantitative characterization of its degradation capability of various lignocellulosic materials and the associated end-products profiles.

## Materials and methods

### Animals and fungal isolation

Twenty white Tianzhu yaks aged 5–6 years with a body weight of 250 ± 20 kg, grazed on wild grass at Wushaoling (37,812–4790°N, 102,851–6950°E; research farm building at 3154 m above sea level) in the Tibetan Autonomous County of Tianzhu (Gansu Province, China) were randomly chosen for fungal isolation. The pasture is alpine meadow with *Polygonum viviparum* and *Kobresia capillifolia* as the main species. The use of animals and the protocol of rumen liquid collection were approved by local farms in the Tibetan Autonomous County and Animal Ethics Committees of Lanzhou University (Gashu, China). Fresh rumen fluids were collected from the rumen of each grazing yak through a stomach tube and were individually inoculated into anaerobic tubes filled with a mixture of 9.0 ml basal liquid medium and 100 mg air-dried chopped wheat straw which was autoclaved at 121 °C for 20 min, supplemented with 1600 IU/ml penicillin and 2000 IU/ml streptomycin. The basal liquid culture medium was modified from that described by Bauchop (1979) and consisted of following components (per liter): yeast extract, 1.0 g; tryptone, 1.0 g; NaHCO_3_, 7.0 g; resazurin 1 mg; l-cysteine hydrochloride, 1.7 g; the rumen fluid of yaks prior to feeding cleared by centrifugation at 10,000×*g* for 20 min at 4 °C, 170 ml; Salt solution I, 165 ml; Salt solution II, 165 ml; and distilled water added to adjust the volume to 1000 ml. Salt solution I contained 6 g/L NaCl, 3 g/L (NH_4_)_2_SO_4_, 3 g/L KH_2_PO_4_, 0.4 g/L CaCl_2_·2H_2_O, and 0.6 g/L MgSO_4_·2H_2_O. Salt solution II contained 4 g/L K_2_HPO_4_.

The anaerobic cultures were incubated at 39 °C and transferred every 4 days without shaking. After several rounds of subculturing, the cultures were serially diluted with the basal liquid medium under anaerobic condition and subsequently inoculated into Hungate roll-tubes that contained molten agar medium supplemented with 0.1% (w/v) glucose. The inoculated agar tubes were promptly rolled in ice water, followed by incubation at 39 °C. The single fungal colonies that formed after 2–3 days’ incubation were picked and inoculated into fresh anaerobic 0.1% (w/v) glucose liquid medium without wheat straw. The procedure was repeated several times between anaerobic liquid glucose medium without wheat straw and agar roll-tubes medium until the fungal colonies on the roll-tubes appeared uniform under the microscope. The cultures were subsequently individually examined for the production of CH_4_ by gas chromatography as described previously (Cheng et al. 2009) to ensure the presence of methanogen. Bacterial contamination in each culture was examined by PCR with the 968f/1401r primer pair (Su et al. 2008). All the cultures were maintained at 39 °C in anaerobic medium with 1% (w/v) wheat straw and transferred every 4 days.

### Fungus and methanogen identification

The co-cultures incubated for 2–3 days in Hungate tubes in 0.1% glucose liquid medium without straw were observed under light microscope. The anaerobic fungi were identified according to the morphological features (Ho and Barr 1995). The co-cultures incubated in 0.1% glucose liquid medium without straw for 4 days were collected by centrifugation at 10,000×*g* at 4 °C for the extraction of the total genomic DNA. The internal transcribed spacer 1 (ITS1) of the anaerobic fungi was amplified with the primer Neo18S For and Neo5.8S Rev as previously described (Edwards et al. 2008). The related ITS1 sequences were deposited in GenBank under accession numbers: KP123392-KP123394. PCR-DGGE (denaturing gradient gel electrophoresis) analysis was used to evaluate the diversity of methanogens in each co-culture, using the 519f/915rGC primers as previously described (Cheng et al. 2009). To identify the methanogens in the co-cultures, the 16S rRNA genes of methanogens were PCR amplified using the Met86F and Met1340R primers (Wright and Pimm 2003) followed by direct sequencing (Beijing Genomics Institute, Beijing, China). The related sequences were deposited in GenBank under accession numbers: KP123413-KP123415. One of the identified (*Piromyces* + *M. ruminantium*) co-cultures, Yak-G18 co-culture, was selected for further characterization. The (*Piromyces* + *M. ruminantium*) Yak-G18 co-culture is available from the authors upon request.

### Scanning electronic microscope

The co-cultures were grown at 39 °C for 72 h in the anaerobic medium containing 100 mg chopped wheat straw and were subsequently collected by centrifugation at 1000×*g* for 5 min. After being washed four times with phosphate-buffered saline (PBS), pH 7.2, the sediments were processed for electron microscopy as described previously (Wei et al. 2016b).

### Experimental design and sampling

For each fermentation experiment, a total of 18 anaerobic bottles were used with each bottle starting with 45 ml basal medium supplemented with 1600 IU/ml penicillin and 2000 IU/ml streptomycin, 5 ml inoculum of co-cultures, and 500 mg of substrate as indicated. The substrates comprise wheat straw (ws), corn stalk (cs), rice straw (rs), Chinese wildrye (cw) and medicago sativa (ms). The fermentation was carried out at 39 °C for 7 days without shaking. During the 7-day incubation, three bottles were taken out every day and analyzed extensively. First, the total gas and methane in the headspace was determined. Then, the cultures were centrifuged at 5000×*g* for 10 min at 4 °C, the resulting supernatants were subject to the analysis of fibrolytic enzyme activities followed by a second centrifugation at 10,000×*g* for 5 min at 4 °C to yield the supernatants subjected to end-product profiling while the resulting pellets were collected for the analysis of IVDMD, NDF and ADF. The medium devoid of inoculum was used as the control. The fermentation experiment was repeated three times.

### Fiber digestibility determination

According to AOAC (1999, ID 930.5**)**, the sample in each bottle was centrifuged and dried at 105 °C for 24 h to determine the in vitro dry matter digestibility (IVDMD). Acid detergent fiber (ADF) and neural detergent fiber (NDF) were determined as described by Van Soest et al. (1991). Alpha amylase was not used, but sodium sulfite was added to each sample for NDF determination. IVDMD or ADFD or NDFD (%) = [(initial DM or ADF or NDF of feed taken for incubation − DM or ADF or NDF of residue at end of incubation)*/*(initial DM or ADF or NDF of feed taken for incubation)] × 100. The DM, ADF and NDF of dried residue from the uninoculated control were taken as initial DM, ADF and NDF, respectively.

### Total gas production and CH_4_ measurement

The total gas production was measured using the pressure transducer technique described by Theodorou et al. (1994). CH_4_ was measured by gas chromatography according to Cheng et al. (2009).

### Determination of enzyme activities

The supernatants were analyzed for the activities of xylanase, filter paperase (FPase), ferulic acid esterase (FAE), acetyl esterase (AE), and *p*-coumaric acid esterase (CAE). The supernatants were diluted 30-fold for the xylanase activity assay, and the xylanase activities were determined at pH 6.8 and 39 °C with substrate solution of 10 g/l birchwood xylan (Sigma, USA). The FPase activities were assayed using Whatman No. 1 filter paper, and detected by a spectrophotometer at 540 nm using the dinitrosalicylic acid method at pH 6.8 and 39 °C as previously described (Lowe et al. 1987). For determining the FAE activities, the supernatants were incubated with methyl ferulate in phosphate buffer solution (pH 6.8) and the absorbances were read with a microplate reader 680XR (Bio-rad, USA) at 340 nm. The FAE activities were subsequently calculated using standard ferulic acid and methyl ferulate curves according to the equation previously published (Yue et al. 2009). The AE activities were assayed in a 96-well reaction microplate by measuring the amount of *p*-nitrophenol released from 1.0 mM *p*-nitrophenyl acetate (Sigma, USA) at pH 6.8 and 39 °C with a micro plate reader 680XR at 415 nm. For assaying the CAE activities, 100 mg de-starched wheat bran were suspended in 1 ml 100 mM MOPS buffer at pH 6.8, followed by incubation with 1 ml supernatant at 39 °C for 20 min. The reaction was terminated by boiling the sample for 3 min, and the *p*-coumaric acid produced was measured by high-performance liquid chromatography (HPLC) (Waters, USA) comprising a Wufeng analytical instrument (Wufeng Co., Ltd, China) and a symmetry reversed-phase C18 column (250 × 4.6 mm, 5 µm, pH 2–8, Waters, USA), according to Wang et al. (2013). The CAE activities were subsequently calculated using the standard curve.

### Determination of ferulic acid and *p*-coumaric acid releases

The levels of ferulic acid and *p*-coumaric acid in the fermentation supernatants were determined by the same method described above for assaying the FAE and CAE activities.

### Analysis of fermentation end-products

One milliliter of supernatant was mixed with 0.3 ml of 250 g/l orthophosphoric acid solution in a polypropylene microtube. The mixture was cooled at 4 °C for 2 h, followed by centrifugation at 15,000×*g* for 10 min at 4 °C. The resulting supernatant was analyzed for acetate, propionate and butyrate levels with HPLC (Agilent 1200, USA) equipped with a ShodexRspak KC-811S-DVB gel col column (Waters, USA) and the SPD-M10AVP detector.

The lactic acid in the supernatant was detected by HPLC (Agilent 1200, USA) equipped with a lactic acid chiral separation column MCI GEL-CRS10W (Mitsubishi Chemical Holdings, Tokyo, Japan) and the SPD-M10AVP detector.

For assaying the succinate level in the supernatant, 5 μl of each sample was separated by ultra-performance liquid chromatography (UPLC) (Waters, USA), followed by measurement with Triple Quadrupole Mass Spectrometer (AB SICEX, USA).

### Statistical analyses

Statistical analyses of the data were performed with SPSS 18.0 software (Microsoft) employing one-way ANOVA followed by Tukey test. All the data were expressed as mean ± SEM and *p* < 0.05 was regarded as statistically significant.

## Results

### Isolation and identification of the fungus–methanogen co-cultures

The fungus–methanogen co-cultures were obtained from the rumen fluids of 20 grazing yaks in Qinghai-Tibetan Plateau. Based on the morphological features and ITS1 region sequences analysis, three of the fungal isolates were classified in the genus *Piromyces* sp. PCR-DGGE analysis (data not shown) indicated that only one methanogenic species exists in each co-culture. This was further verified by 16S rRNA sequencing which identified all the methanogens in the three co-culture isolates as *M. ruminantium*. We subsequently denoted these three fungus–methanogen co-cultures as (*Piromyces* + *M. ruminantium*) Yak-G18 to Yak-G20.

**Fig. 1:**
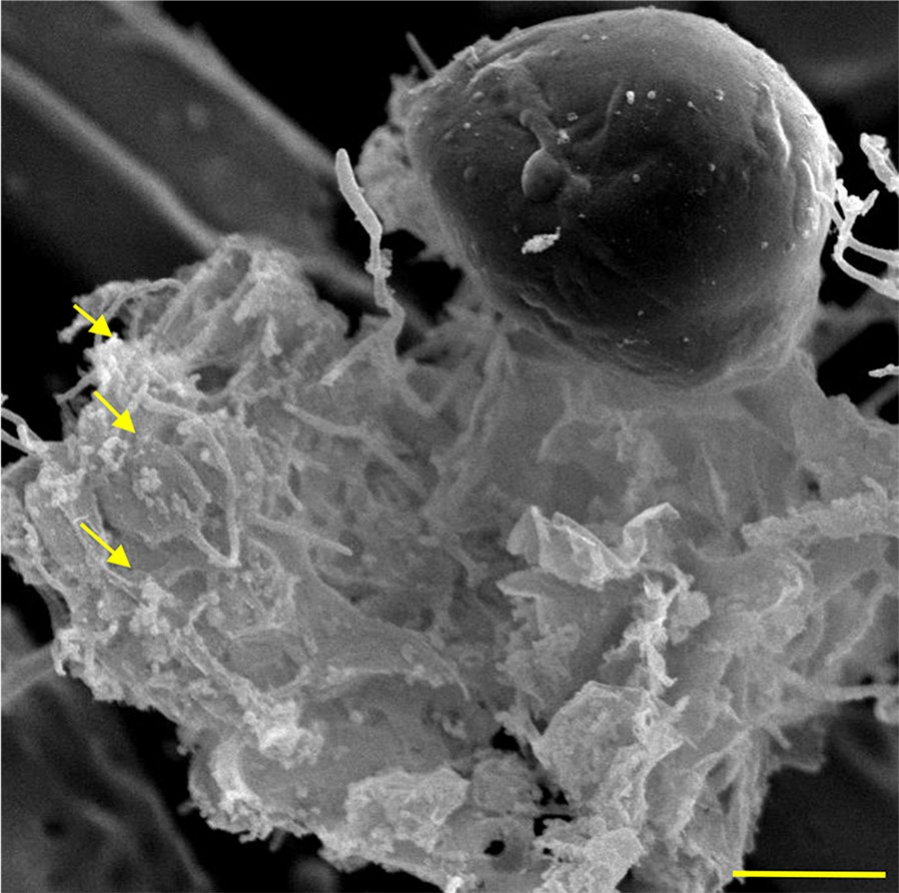
Scanning electron micrograph of the (*Piromyces* + *M. ruminantium*) Yak-G18 co-culture grown on wheat straw. *Arrow, M. ruminantium*; *bar* 10 μm

Among the three (*Piromyces* + *M. ruminantium*) co-cultures, (*Piromyces* + *M. ruminantium*) Yak-G18 co-culture exhibits the most rapid growth on Hungate agar roll-tubes and the fastest time to float up straw in anaerobic liquid medium. Thus, we selected (*Piromyces* + *M. ruminantium*) Yak-G18 co-culture for further characterization. We first analyzed (*Piromyces* + *M. ruminantium*) Yak-G18 co-culture by scanning electronic microscopy, and the result showed the rhizoids as the main fungal attachment sites of methanogen (Fig. 1).

### Fiber digestion capability assay

**Fig. 2:**
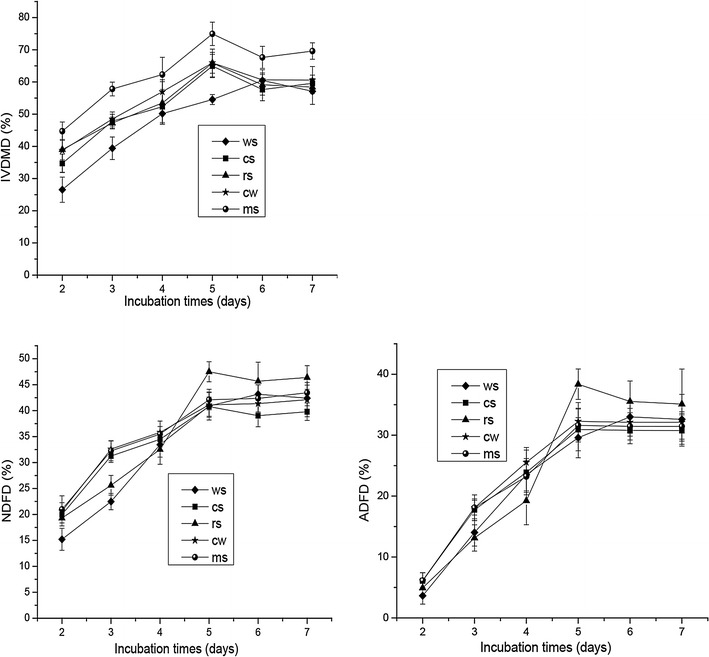
Fiber degradation by the (*Piromyces* + *M. ruminantium*) Yak-G18 co-culture during the 7 days’ incubation (*IVDMD* in vitro dry matter digestibility, *ADFD* acid detergent fiber digestibility, *NDFD* neural detergent fiber digestibility, wheat straw *diamond*, corn stalk *square*, rice straw *triangle*, Chinese wildrye *star*, medicago sativa *circle*)

**Fig. 3:**
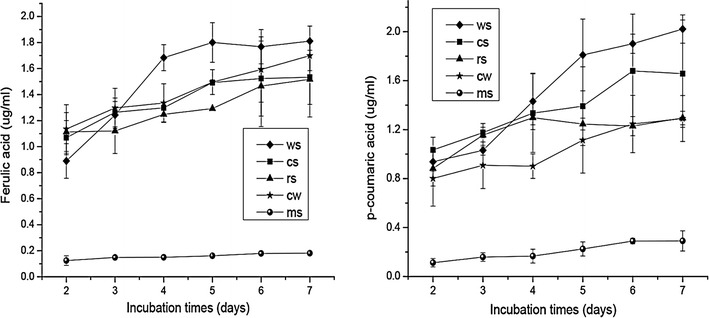
Releases of ferulic acid and *p*-coumaric acid by the (*Piromyces* + *M. ruminantium*) Yak-G18 co-culture grown on chopped wheat straw, corn stalk, rice straw, Chinese wildrye and medicago sativa during the 7 days’ incubation (wheat straw *diamond*, corn stalk *square*, rice straw *triangle*, Chinese wildrye *star*, medicago sativa *circle*)

We determined the fermentation activities of (*Piromyces* + *M. ruminantium*) Yak-G18 co-culture with various lignocellulose substrates (Table 1), which include wheat straw, corn stalk, rice straw, Chinese wildrye, and medicago sativa, during a 7 days’ incubation. During the incubation, (*Piromyces* + *M. ruminantium*) Yak-G18 co-culture degraded 60.5% of wheat straw, 65.0% of corn stalk, 65.9% of rice straw, 66.0% of Chinese wildrye, 75.0% of medicago sativa, respectively (Fig. 2). The digestion kinetics on the five substrates shared common features: dry matter was rapidly lost from day 2 to day 3, so were the acid detergent fiber (ADF) and the neural detergent fiber (NDF). At approximately day 5 or day 6, the peak ADFD values (30.9–38.4%) and NDFD values (40.8–47.5%) reached (Fig. 2). With all the five substrates, fermentation by (*Piromyces* + *M. ruminantium*) Yak-G18 co-culture leaded to the release of low, but detectable levels of ferulic acid (0.2–1.8 µg/ml) and *p*-coumaric acid (0.3–2.0 µg/ml) (Fig. 3). Among the substrates, the medicago sativa showed the lowest release level of ferulic acid and *p*-coumaric acid, consistent with it having the lowest contents of both acids (Table 1).

**Table 1:** Composition of the forages used as substrates before incubation (g/kg DM)

	Wheat straw	Corn stalk	Rice straw	Chinese wildrye	Medicago sativa
Dry matter	939.57	940.05	953.40	939.33	952.40
Crude protein	34.10	47.89	49.29	87.81	203.82
Crude fat	7.93	21.67	4.63	8.00	9.00
NDF	782.94	752.41	717.3	752.82	426.07
ADF	503.53	426.29	444.69	408.65	324.91
Ash	108.59	65.11	124.24	54.49	106.82
Ferulic acid	3.26	7.53	6.06	6.40	1.50
*p*-Coumaric acid	5.04	14.71	7.26	6.22	1.08

### Cumulative gas production

**Fig. 4:**
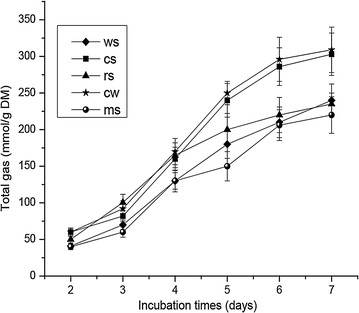
Cumulative gas production by the (*Piromyces* + *M. ruminantium*) Yak-G18 co-culture grown on chopped wheat straw, corn stalk, rice straw, Chinese wildrye and medicago sativa during the 7 days’ incubation (wheat straw *diamond*, corn stalk *square*, rice straw *triangle*, Chinese wildrye *star*, medicago sativa *circle*)

Our measurement of the total gas production was consistent with effective fiber degradation. With all the five substrates, fermentation by (*Piromyces* + *M. ruminantium*) Yak-G18 co-culture exhibited similar gas production kinetics that features a constant accumulation of gas throughout the 7 days’ incubation (Fig. 4). The values of total gas production at the end of incubation ranged between 220 ml/g DM (medicago sativa) and 309 ml/g DM (Chinese wildrye).

### Fibrolytic enzyme profiles

**Fig. 5:**
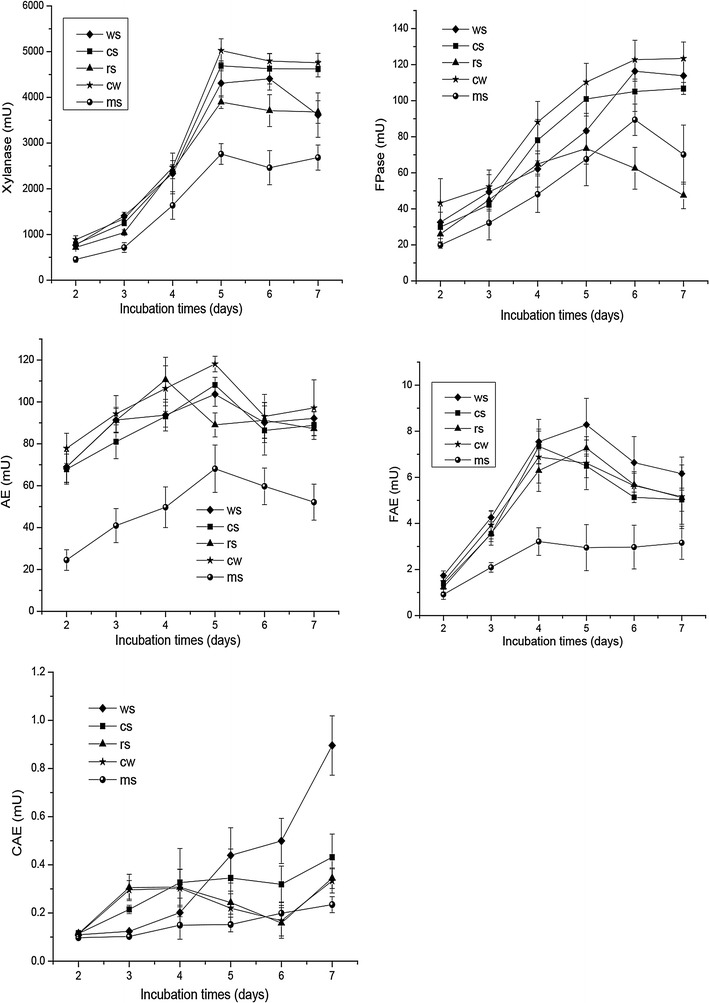
Fibrolytic profiles of the (*Piromyces* + *M. ruminantium*) Yak-G18 co-culture grown on chopped wheat straw, corn stalks, rice straw, Chinese wildrye and medicago sativa during the 7 days’ incubation (*FPase* filter paper ase, *FAE* ferulic acid esterase, *CAE p*-coumaric acid esterase, *AE* acetyl esterase, wheat straw *diamond*, corn stalk *square*, rice straw *triangle*, Chinese wildrye *star*, medicago sativa *circle*)

We further investigated the effects of different fermentation substrates (Table 1) on the profile of fibrolytic enzymes produced by (*Piromyces* + *M. ruminantium*) Yak-G18 co-culture. Specifically, we measured the activities of filter paper cellulase (FPase), xylanase, acetyl esterase (AE), ferulic acid esterase (FAE), and *p*-coumaric acid esterase (CAE). As shown in Fig. 5, the overall fibrolytic enzyme profiles were quite similar among all the five substrates. Xylanase was the most prominent fibrolytic enzyme with maximal enzymatic activity ranging from 2750 mU (medicago sativa) to 5023 mU (Chinese wildrye). Significant levels of FPase and AE were also detected with peak values ranging from 71.9–123.5 mU (from rice straw to Chinese wildrye) and 66.3–118.1 mU (from medicago sativa to Chinese wildrye) respectively. During the 7 days’ incubation, the activities of xylanase, FPase and AE were continuously accumulated in the initial phase and reached maximum levels at day 5 for xylanase or day 6 for FPase and AE except on rice straw that day 4 was marked with the presence of highest FPase activity. In contrast, only very low FAE and CAE activities were detected, both showing the highest levels on wheat straw with respective values of 8.3 and 0.9 mU, which is consistent with the low release of ferulic acid and *p*-coumaric acid as shown in Fig. 3.

### End-products generation

A remarkable characteristic of fungus–methanogen co-culture that differs from fungus culture is that methanogen can utilize fungal metabolites including H_2_, CO_2_, formate to produce methane, and consequently tilt the fungal metabolism toward synthesis of more ATP and acetate (Bauchop and Mountfort 1981; Marvin-Sikkema et al. 1993). Consistent with this characteristic, after the 7 days’ incubation, (*Piromyces* + *M. ruminantium*) Yak-G18 co-culture yielded significant amount of CH_4_ on all the five substrates with values ranging from 1.3 mmol/g DM on medicago sativa to 2.4 mmol/g DM on wheat straw (Table 2). It also produced high levels of acetate with the final concentrations of 24.1, 39.6, 32.0, 41.3, 36.9 mM on wheat straw, corn stalk, rice straw, Chinese wildrye and medicago sativa, respectively (Table 2). Contrastingly, it exhibited little production of succinate, further corroborating the profound impact of methanogen on fungal metabolism. The other main end product, lactate, was significantly produced by (*Piromyces* + *M. ruminantium*) Yak-G18 co-culture with all the substrates. Of note, such lactate production mainly consists of l-lactate at levels between 3.6 mM (rice straw) and 5.8 mM (corn stalk) which were many times higher than the corresponding levels of d-lactate.

**Table 2:** End-products produced by the (*Piromyces* + *M. ruminantium*) Yak-G18 co-culture grown on chopped wheat straw, corn stalk, rice straw, Chinese wildrye and medicago sativa during the 7 days’ incubation

End-products	(*Piromyces* + *M. ruminantium*) Yak-G18 co-culture						
	2 days	3 days	4 days	5 days	6 days	7 days	Average
Acetate (mM)							
ws	9.23^d^	12.36^c^	20.56^b^	23.10^b^	24.13^d^	23.36^c^	18.79^a^
cs	15.16^ab^	22.24^ab^	29.27^a^	31.04^a^	37.07^ab^	39.60^a^	29.07^ab^
rs	11.34^cd^	15.41^bc^	20.34^b^	24.55^b^	30.23^c^	32.01^b^	22.31^c^
cw	16.83^a^	23.24^a^	29.94^a^	32.37^a^	38.41^a^	41.27^a^	30.34^a^
ms	13.50^bc^	21.57^b^	28.61^a^	30.37^a^	33.74^bc^	36.94^ab^	27.45^b^
CH_4_ (mmol/g DM)							
ws	0.52^b^	0.86^a^	1.27^a^	1.44^ab^	2.33^a^	2.35^a^	1.46^a^
cs	0.74^a^	0.86^a^	1.31^a^	1.55^a^	1.86^ab^	1.90^b^	1.37^b^
rs	0.60^ab^	0.96^a^	1.16^a^	0.93^c^	1.42^bc^	1.62^bc^	1.11^c^
cw	0.78^a^	0.89^a^	1.44^a^	1.65^a^	1.86^ab^	1.96^b^	1.43^ab^
ms	0.61^ab^	0.79^a^	1.14^a^	1.20^bc^	1.30^c^	1.28^c^	1.05^c^
d-lactate (mM)							
ws	0.15^a^	0.19^a^	0.24^a^	0.24^a^	0.24^a^	0.23^a^	0.22^a^
cs	0.12^a^	0.15^a^	0.16^b^	0.16^b^	0.16^a^	0.17^ab^	0.15^b^
rs	0.14^a^	0.16^a^	0.19^ab^	0.21^ab^	0.21^a^	0.22^a^	0.19^ab^
cw	0.12^a^	0.14^a^	0.13^b^	0.16^b^	0.16^a^	0.15^b^	0.14^b^
ms	0.13^a^	0.16^a^	0.17^b^	0.16^b^	0.21^a^	0.21^ab^	0.17^ab^
l-lactate (mM)							
ws	3.86^a^	4.41^a^	4.98^a^	5.22^ab^	5.54^a^	4.86^a^	4.81^a^
cs	3.92^a^	4.32^ab^	5.52^a^	5.79^a^	5.76^a^	4.89^a^	5.03^a^
rs	3.26^a^	3.26^b^	3.50^b^	3.56^b^	3.29^b^	3.57^a^	3.40^b^
cw	3.72^a^	4.19^ab^	5.42^a^	5.73^a^	5.63^a^	4.79^a^	4.91^a^
ms	3.59^a^	3.99^ab^	5.19^a^	5.46^ab^	5.10^ab^	4.55^a^	4.64^a^
Lactate (mM)							
ws	4.02^a^	4.60^a^	5.21^a^	5.45^ab^	5.78^a^	5.09^a^	5.03^a^
cs	4.04^a^	4.47^a^	5.68^a^	5.93^a^	5.93^a^	5.06^a^	5.19^a^
rs	3.40^a^	3.42^a^	3.69^b^	3.77^b^	3.49^b^	3.80^a^	3.59^b^
cw	3.84^a^	4.32^a^	5.55^a^	6.01^a^	5.79^a^	4.94^a^	5.05^a^
ms	3.72^a^	4.14^a^	5.36^a^	5.62^ab^	5.31^ab^	4.76^a^	4.82^ab^
Succinate (mM)							
ws	0.02^a^	0.04^ab^	0.05^a^	0.05^a^	0.06^ab^	0.06^ab^	0.05^a^
cs	0.03^a^	0.04^ab^	0.05^a^	0.06^a^	0.07^a^	0.08^a^	0.05^a^
rs	0.03^a^	0.03^b^	0.04^a^	0.05^a^	0.05^b^	0.05^b^	0.04^a^
cw	0.04^a^	0.05^a^	0.06^a^	0.06^a^	0.08^a^	0.08^a^	0.06^a^
ms	0.03^a^	0.03^b^	0.05^a^	0.07^a^	0.08^a^	0.08^a^	0.06^a^

Means in a row without same superscript letter differ as noted *p* value

*SEM* standard error of means (n = 3), *ws* wheat straw, *cs* corn stalk, *rs* rice straw, *cw* Chinese wildrye, *ms* medicago sativa

## Discussion

Anaerobic fungi reside in the rumen of herbivore and synergize with associated methanogenic archaea to decompose ingested plant biomass. Given the relative short retention time of lignin tissues in the rumen, researchers have long speculated that rumen fungus–methanogen consortiums have acquired potent lignocellulose degradation capability attributable to natural selection. Thus, natural fungus–methanogen co-cultures may have the potential to be directly used in industrial bioconversion of renewable lignocellulosic biomass. Of equal importance, studies on these co-cultures may provide new insights into mechanistic aspects of fungal lignocellulolytic machinery, therefore facilitating the development of lignocellulolytic enzyme mixtures or complexes with improved biodegradation efficiency.

Despite their potential importance in bioconversion industry, natural fungus–methanogen co-cultures are still poorly understood, largely attributable to the fact that only a few of them were isolated. A total of only ten natural fungus–methanogen co-cultures had been previously isolated from mule, camel, buffalo, goat, sheep and Alpine ibex (Bauchop and Mountfort 1981; Jin et al. 2011; Leis et al. 2014). Our search for highly active natural fungus–methanogen co-cultures has been focusing on yaks grazed on the Qinghai-Tibetan Plateau at 3000 m elevation and above. The underlying rationale can be ascribed to the supposition that the adaptation of yak to extreme environment drives the evolution of its rumen microbial community to be superior in decomposing recalcitrant lignocellulosic polymers.

In this study, we investigated three *Piromyces* with methanogen co-cultures isolated from the rumen liquid of grazing yaks. Like most previously reported natural fungus–methanogen co-cultures from the rumen or feces of other herbivores (Bauchop and Mountfort 1981; Jin et al. 2011), all the three co-culture isolates showed the one fungus–one methanogen pattern. All the methanogens associated with the three isolates were identified to be *Methanobrevibacter ruminantium*, consistent with previous finding that *Methanobrevibacter ruminantium* is one of the most abundant H_2_- and CO_2_-consuming rumen methanogen species (Carberry et al. 2014; Janssen and Kirs 2008). Electron microscopy analysis of the representative co-culture isolate, (*Piromyces* + *M. ruminantium*) Yak-G18 co-culture, unveiled physical interactions between the methanogen and rhizoids but not the sporangia surface of the fungus, reminiscent of the majority of previously reported natural fungus–methanogen co-cultures (Bauchop and Mountfort 1981; Jin et al. 2011). Such close proximity between fungus and methanogen might facilitate effective interspecies hydrogen transfer (Leschine 1995; Thareja et al. 2006).

Our comprehensive, quantitative analysis of the fermentation activities of (*Piromyces* + *M. ruminantium*) Yak-G18 co-culture on various substrates support our original hypothesis that Yak-derived ruminal fungus–methanogen consortiums have evolved to possess high efficiency to degrade plant lignocellulose. Given that the IVDMD values are often fluctuated at the late time points of the fermentation due to the outgrowth of the fungi, we also employed ADFD and NDFD values for accurately measuring the biodegrading activities. (*Piromyces* + *M. ruminantium*) Yak-G18 co-culture showed higher fiber digestion capability than most, if not all, of the previously reported anaerobic fungus isolates from other herbivores (Jin et al. 2011; Paul et al. 2010; Thareja et al. 2006). Specifically, the wheat straw IVDMD values of (*Piromyces* + *M. ruminantium*) Yak-G18 co-culture are significantly higher than those of many natural anaerobic fungus cultures isolated from various herbivores (Paul et al. 2010; Thareja et al. 2006). The 4-day wheat straw IVDMD and rice straw IVDMD values of (*Piromyces* + *M. ruminantium*) Yak-G18 co-culture are also slightly higher than those of the two previously reported natural fungus–methanogen co-cultures also with *Piromyces* as the fungal constituent (Jin et al. 2011). Consistent with its high IDVMD values, (*Piromyces* + *M. ruminantium*) Yak-G18 co-culture also exhibits generally high ADFD and NDFD values of all five substrates tested, further confirming its great lignocellulose degradation capability.

Strong fiber degradation capability is normally associated with the production of highly active fibrolytic enzymes. As expected, (*Piromyces* + *M. ruminantium*) Yak-G18 co-culture showed high yield of a number of fibrolytic enzyme activities, especially xylanase activity, during the incubation with all the five lignocellulosic substrates assayed. Notably, it showed significantly higher xylanase activity (2500–5023 mU maximal value) than numerous fungus isolates of various herbivore origins (Cao and Yang 2011; Sirohi et al. 2013; Thareja et al. 2006; Tripathi et al. 2007). In comparison to the xylanase activity, the FPase and AE activities of the (*Piromyces* + *M. ruminantium*) Yak-G18 co-culture are relatively lower while the feruloyl esterase activities were the least significant, irrespective of the substrates. This result is corroborated by a most recent study that a gut *Piromyces* isolate exhibits approximately a fourfold increase of lignocellulose-induced xylanase activity relative to commercial Aspergillus enzyme preparations (Solomon et al. 2016). Thus, (*Piromyces* + *M. ruminantium*) Yak-G18 co-culture can be classified as a new practical agent for industrial xylanase production.

There is accumulated evidence that the production of fibrolytic enzymes by anaerobic fungus is highly affected by growth conditions (Glass 2016; Solomon et al. 2016). Consistent with this notion, we found that different lignocellulosic materials induced different levels of the fibrolytic enzymes produced by (*Piromyces* + *M. ruminantium*) Yak-G18 co-culture. Among the five assayed substrates, medicago sativa which possesses the least NDF (Table 1) was identified with the highest IVDMD value. In contrast, compared with other substrates, medicago sativa showed least efficacy in inducing xylanase as well as AE activities throughout the incubation period. The induction of FPase by medicago sativa was also lower than other substrates except for Chinese wildrye at late incubation phase. The actual relationship between the fibrolytic enzyme production of (*Piromyces* + *M. ruminantium*) Yak-G18 co-culture and the fiber composition of fermented material requires future investigations.

The advantage of anaerobic fungus–methanogen co-culture over fungus culture in degrading lignocellulose stems from the ability of methanogen to utilize H_2_ and CO_2_ for methane production, thus eliminating the inhibition of these end-products on the fungal growth and consequently the associated enzyme activities (Marvin-Sikkema et al. 1993; Williams et al. 1994). Indeed, for all the assayed substrates, the (*Piromyces* + *M. ruminantium*) Yak-G18 co-culture displayed a continuous accumulation of CH_4_, the levels of which were significantly higher than those of the natural fungus–methanogen co-cultures reported by Jin et al. (2011). One well-known metabolic impact of methanogen association on anaerobic fungus is the enhanced hydrogenosome pathway leading to more production of acetate and ATP (Williams et al. 1994). Consistent with this notion, (*Piromyces* + *M. ruminantium*) Yak-G18 co-culture produced high levels of acetate on all five assayed substrates with maximal value of 41.3 mM with Chinese wildrye on day 7. With regard to the acetate yield, a direct comparison between (*Piromyces* + *M. ruminantium*) Yak-G18 co-culture and natural fungus–methanogen co-cultures reported by Jin et al. (2011) is not valid because of different substrates being used for the determinations.

End-products profiling also revealed a unique metabolism feature of the (*Piromyces* + *M. ruminantium*) Yak-G18 co-culture as the high-yield production of lactate. Significant lactate production has only been associated with some natural fungus–methanogen co-cultures isolated so far (Jin et al. 2011). Compared with these lactate-producing co-cultures previously reported (Jin et al. 2011), (*Piromyces* + *M. ruminantium*) Yak-G18 co-culture yields approximately four to sevenfold more lactate with l-lactate being the predominant isoform. It is worth mentioning that, since lactate cannot be metabolized by methanogen, the association with methanogen could impact the fungal lactate production either positively or negatively. It is as yet unclear why (*Piromyces* + *M. ruminantium*) Yak-G18 co-culture produces much more l-lactate than d-lactate and the underlying mechanism awaits further investigation.

Finally, the identification of (*Piromyces* + *M. ruminantium*) Yak-G18 co-culture extends the repertoire of Yak-derived ruminal fungus–methanogen co-cultures applicable to the biodegradation industry. Previously, we isolated and identified two other types of fungus–methanogen co-cultures from yaks grazing on the Qinghai-Tibetan Plateau, namely (*Orpinomyces joyonii* + *M. ruminantium*) type, and the (*Neocallimastix frontalis* + *M. ruminantium*) type (Wei et al. 2016b). Among the three types of co-cultures, based on the data presented here and our previous studies (Wei et al. 2016a, b), we concluded that (*Piromyces* + *M. ruminantium*) Yak-G18 co-culture and the representative member of (*N. frontalis* + *M. ruminantium*) type, (*N. frontalis* + *M. ruminantium*) Yaktz1 co-culture, have an advantage over the nine members of (*Orpinomyces joyonii* + *M. ruminantium*) type in degrading lignocellulosic materials, as indicated by higher IVDMD values along with higher fibrolytic enzyme activities. Besides, the two co-cultures also exhibits higher yield of CH_4_ and acetate, demonstrating a stronger metabolic fungus–methanogen syntrophy that is manifested by the fact that (*Piromyces* + *M. ruminantium*) Yak-G18 co-culture grew very stably in anaerobic medium with wheat straw and showed constant production of end-products even after 2 years’ continuous sub-culturing.

It should be emphasized here that *Piromyces* and *Neocallimastix* have been shown to have growth advantages on plant lignocellulose over other anaerobic fungal genera (Paul et al. 2010). Most recently, Solomon et al. (2016) characterized three gut anaerobic fungi including *Piromyces* and *Neocallimastix* by transcriptome and proteome approaches. The analysis revealed that these gut fungi, particularly *Piromyces*, possess a wide array of cellulolytic carbohydrate-active enzymes (CAZy) with diverse functions in deconstructing cellulose and hemicellulose. The expansion of CAZy gene family, especially the xylan-degrading enzymes, provides a plausible explanation why *Piromyces* secretion showed limited substrate preference as compared to the commercial mixtures produced by *Trichoderma* and *Aspergillus*. This elegant study further endorses the candidacy of (*Piromyces* + *M. ruminantium*) Yak-G18 co-culture as a new model platform for developing more efficient fibrolytic enzyme formula in lignocelluloses degradation industry.

In conclusion, three *Piromyces* with *M. ruminantium* co-cultures were obtained from the rumen contents of the yaks grazing on the Qinghai-Tibetan Plateau. The representative co-culture, (*Piromyces* + *M. ruminantium*) Yak-G18 co-culture, displayed robust lignocellulose degradation capability on various substrates by exhibiting high IVDMD, NDFD, and ADFD, along with the production of high levels of xylanase and other fibrolytic enzyme activities. The end-products profiling of (*Piromyces* + *M. ruminantium*) Yak-G18 co-culture validated the fungus–methanogen syntrophy, as is demonstrated by the high-yield production of methane and acetate and sustained growth on lignocellulose. These results, corroborated by recent advances in transcriptome and proteome analysis of anaerobic gut fungi, strongly support that (*Piromyces* + *M. ruminantium*) Yak-G18 co-culture and its future exploration might be of great use to lignocellulose biotechnology.

## Abbreviations

ADFD: acid detergent fiber digestibility
AE: acetyl esterase
CAE: coumaric acid esterase
CTAB: hexadecyl trimethyl ammonium bromide
DGGE: denatured gradient gel electrophoresis
DNS: 3,5-dinitrosalicylic acid
ESI: electrospray native ionization
FA: ferulic acid
FAE: ferulic acid esterase
FPase: filter paper ase
HPLC: high-performance liquid chromatography
ITS1: internal transcribed spacer region 1
IVDMD: in vitro dry matter disappearance
LC-MS: liquid chromatograph mass spectrometer
MOPS: 3-morpholinopropane sulfonic acid
MRM: multiple reaction monitoring
NDFD: neutral detergent fiber digestibility
PBS: phosphate buffer saline
PCA: *p*-coumaric acid
UPLC: ultra performance liquid chromatography
